# Pseudo-Sample Generation and Self-Supervised Framework for Infrared Dim and Small Target Detection

**DOI:** 10.3390/e27121212

**Published:** 2025-11-28

**Authors:** Jinxin Guo, Weida Zhan, Dehua Huo, Depeng Zhu, Yu Chen, Yichun Jiang, Xiaoyu Xu

**Affiliations:** School of Electronic and Information Engineering, Changchun University of Science and Technology, Changchun 130022, China

**Keywords:** information processing, target detection, pseudo-sample generation, image degradation, self-supervised learning

## Abstract

Infrared dim and small target detection is crucial for long-range sensing. However, its deep representation learning is severely constrained by the scarcity of accurately annotated real data, and related research remains underdeveloped. Existing data generation methods based on patch synthesis or geometric transformations fail to incorporate the physical degradation mechanisms of infrared imaging systems and reasonable environmental constraints, leading to significant discrepancies between synthetic data and real-world scenarios. To address this issue, this paper proposes a novel pseudo-sample generation paradigm based on physics-informed degradation modeling and high-order constraints. First, we construct an infrared image degradation model that decouples the degradation processes of targets and backgrounds at the signal level, achieving accurate modeling of real infrared imaging while ensuring the reliability of the degradation process through information fidelity optimization. Second, an online grid-based high-order constraint strategy is designed, which synergistically integrates global semantic, local structural, and grayscale constraints based on statistical distribution consistency to generate a high-fidelity infrared simulation dataset. Finally, we build a complete self-supervised detection framework incorporating classical neural networks, customized loss functions, and two-dimensional information evaluation metrics. Extensive experiments demonstrate that the synthetic data generated by our method significantly outperforms existing simulated datasets on authenticity metrics. It also effectively enhances the generalization performance of various detectors in real-world scenarios, achieving detection accuracy superior to baseline models trained on traditional simulated data.

## 1. Introduction

Infrared dim and small target detection serves as one of the core technologies in information processing fields such as infrared early warning, precision guidance, and remote monitoring [1,2,3,4]. However, infrared dim and small targets in natural scenes typically exhibit extremely low signal-to-noise ratios and information entropy, lack distinct texture information and shape features, and are susceptible to interference from complex backgrounds. These factors pose severe challenges to the detection task. Although deep learning methods have achieved remarkable success in visible light object detection, their application in infrared dim and small target detection is severely constrained by the scarcity of high-quality annotated data. Acquiring large-scale, diverse real infrared images along with pixel-level annotations is not only costly but also nearly infeasible in sensitive domains such as defense, constituting a primary bottleneck for the development of this field.

To overcome this data bottleneck, using synthetic data to train detection models has emerged as a promising alternative. Traditional methods based on simple patch pasting or geometric transformations [5,6] can expand data volume to some extent, but their core flaw lies in the failure to embed the physical mechanisms of infrared imaging systems. The pseudo-samples generated by these methods show significant differences from real physical processes in terms of degradation characteristics, radiation properties, and target–background interactions, leading to severe performance degradation when models trained on such data are generalized to real complex scenarios.

**Figure 1:**
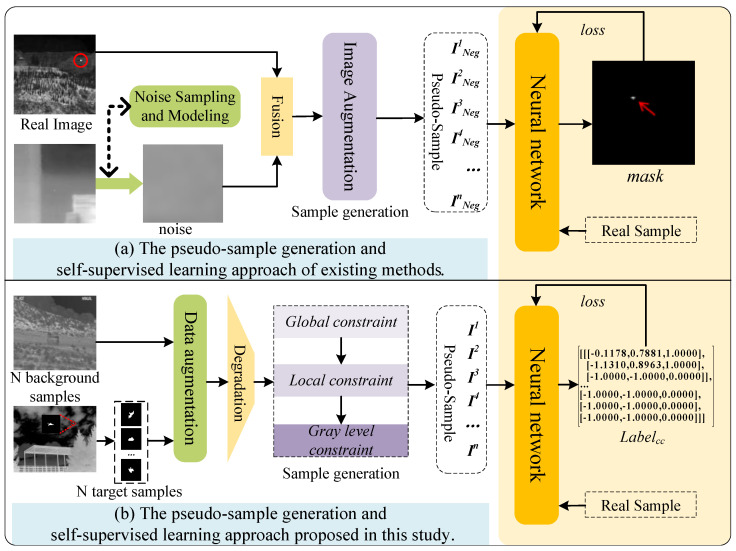
Comparison between the proposed method and existing pseudo-sample generation and network learning approaches. Section (**a**) shows existing methods, while section (**b**) presents our proposed framework. The proposed online pseudo-sample generation method simulates real image degradation mechanisms and incorporates high-order constraints, resulting in generated targets that exhibit closer alignment with real images in terms of quantity, spatial distribution, and information characteristics.

In recent years, the development of pseudo-sample generation algorithms for real infrared dim and small targets has remained largely stagnant. However, several existing heuristic data synthesis methods have provided important references and insights for this study. As shown in Figure 1, current pseudo-sample generation methods start from real infrared images and employ noise sampling and modeling techniques combined with hybrid enhancement strategies to produce pseudo-samples for network training. Although these approaches achieve certain effectiveness in expanding data volume, they fundamentally rely on statistical resampling and noise injection of existing real images. They lack in-depth modeling of information entropy and feature distributions, failing to deeply capture the intrinsic degradation mechanisms between targets and backgrounds in infrared imaging at a physical level. This limitation consequently restricts the generalization performance of detection models trained on such data.

In addition, several other representative methods have explored pseudo-sample generation from different perspectives. Sun et al. [7] proposed a generation method based on physical priors in the RDIAN network, which uses a Gaussian sphere model to simulate the radiation characteristics of targets and integrates them with real collected infrared background images while introducing sensor-specific noise. The ISD-DCGAN method proposed by Zhang et al. [6] explores the application of generative adversarial networks in infrared sequence data generation. This approach decouples the processes of background generation, target generation, and sequence construction through an improved deep convolutional generative adversarial network, attempting to ensure visual realism and sequence continuity of generated samples through adversarial training. However, its integration of infrared physical mechanisms remains relatively indirect, focusing more on data distribution matching. In the LASNet study, Chen et al. [8] further proposed the DISTG algorithm for pseudo-sample generation targeting dense objects. This method models the spatiotemporal distribution and thermodynamic interactions of target clusters through physical simulation, demonstrating its distinctive strength in simulating dense, moving small targets. Nevertheless, its complexity is relatively high, and the modeling of degradation details for targets and backgrounds in single-frame static images could be further refined.

In summary, although existing data synthesis methods have made certain progress, they generally exhibit deficiencies in the depth of physical degradation information modeling and the strength of constraints in the generation process. Specifically, most of these methods remain at the level of appearance enhancement or the introduction of partial physical parameters, failing to systematically construct a complete degradation model. They also lack the application of multi-level constraints that integrate global and local coordination to ensure the physical authenticity and distributional rationality of the generated samples.

To address the aforementioned challenges, this study focuses on the critical issue of high-fidelity infrared pseudo-sample generation, aiming to enhance the quality of synthetic data from both physical degradation and data information distribution perspectives. We contend that effective simulation of infrared dim and small targets must satisfy three requirements: first, establishing an accurate imaging degradation model that separately characterizes the radiation and noise properties of targets and backgrounds; second, adhering to the spatial information distribution patterns of targets in real scenarios, such as occurrence density and positional preferences; and third, ensuring the generated data can effectively drive the detection model to learn feature representations with generalization capability, rather than overfitting to simulation artifacts. Based on this, we propose an information-driven physical degradation framework for high-fidelity infrared dim and small target pseudo-sample generation and self-supervised detection, providing an important solution to address the issues of data scarcity and reliability verification in this field.

In summary, the contributions of this paper can be summarized as follows:

- We propose a real physics-driven infrared image degradation model, termed TOA, which decouples the degradation processes of targets and backgrounds at the signal level, achieving accurate modeling of the real infrared imaging process.
- We propose an online grid-based high-order constraint pseudo-sample generation method. This method constrains the position, quantity, and authenticity of dim and small targets in generated pseudo-sample images, bridging the information gap between pseudo-samples and real samples.
- We construct a complete self-supervised learning detection framework. For the customized pseudo-labels, we designed a dedicated novel position-confidence loss function and a position deviation rate evaluation metric.

## 2. Related Work

### 2.1. Infrared Dim and Small Target Detection

The technology of infrared dim and small target detection has undergone a paradigm shift from traditional model-driven approaches to modern data and information-driven methods. Traditional approaches primarily relied on handcrafted features or prior models, such as filter-based methods [9], human visual system-based methods [10], and low-rank matrix recovery-based methods [11]. These methods achieve target detection by constructing complex target-background separation models. However, their performance heavily depends on the accuracy of the preset models, resulting in limited generalization capability in complex and variable real-world scenarios.

With breakthroughs in deep learning, data and information-driven detection methods have demonstrated significant advantages. Based on the evolution of network architectures, existing deep learning methods can be broadly categorized into two main streams: convolutional neural network (CNN)-based methods and Transformer-based methods.

CNN-based methods aim to address the conflict between clutter suppression and feature information retention. Existing work, such as ISTC [12], introduces a context modulation mechanism focusing on enhancing the correlation between target pixels to mitigate clutter effects, yet its background suppression capability remains incomplete. To improve this issue, densely nested networks [13,14] combined with attention mechanisms attempt to preserve deep target semantics by deepening the network structure and strengthening cross-layer feature interactions, but they fail to fundamentally resolve the class imbalance between foreground targets and background clutter. Addressing this, RDIAN [7] utilizes multi-directional convolutional layers to extract target features and dynamically balances the feature responses of targets and backgrounds through progressive interactions within receptive fields, enhancing the model’s discriminative ability. Furthermore, networks based on the U-Net backbone, such as UIU-Net [15], UCDnet [16], and MSHNet [17], have become mainstream. Transformer-based methods (ISTT [18,19]), on the other hand, leverage self-attention mechanisms to model long-range dependencies in images, overcoming the limitations of local receptive fields in CNNs.

Nevertheless, existing deep learning methods heavily rely on large amounts of accurately annotated data, and the scarcity of such data has become a major bottleneck for further performance improvement.

### 2.2. Sample Generation

Pseudo-sample generation represents the most critical step in applying self-supervised learning to infrared dim and small target detection [20,21,22]. The selection of positive target samples and negative background samples directly determines the authenticity of data information in pseudo-samples. Positive target samples can be obtained through two methods: The first is simulation-based generation, where each generated positive sample image must undergo strict constraints to maintain a feature distribution consistent with real positive sample images. Crucially, simulated positive target samples should cover different target sizes, shapes, and intensity characteristics to ensure the model learns truly useful target features. The alternative method involves first acquiring image samples containing small targets and then extracting real positive target samples from them. This study combines both approaches, generating a total of 2929 positive sample images for final sample generation.

The most direct method for obtaining negative background samples is field photography, which follows three principles [23]: (1) Multi-scenario diversity: Ensuring diversity of negative background images by covering different environments and scenario conditions, with random variations in location, weather, time, and season to obtain diverse negative background samples. (2) Distribution balance: Maintaining balanced selection of negative background images to avoid excessive bias toward any particular background type. A balanced negative background dataset helps the model better distinguish between targets and backgrounds. (3) Interference-free: Ensuring background images contain no sharp interfering elements or non-target objects, preventing the network from learning incorrect behaviors when encountering unlabeled interfering targets. This study collected 21,419 negative background images covering multiple scenarios including clouds, buildings, forests, ridges, pedestrians, and vehicles. The obtained negative background samples are highly diverse, essentially covering all common scenarios encountered in daily environments.

### 2.3. Image Degradation Model

No imaging system in the real world is perfect; all are affected by various image degradation mechanisms [24], which underscores the necessity of researching image enhancement and information processing technologies. Therefore, the target positive sample images and background negative sample images obtained in this study cannot be directly used for infrared small target pseudo-sample synthesis. They must undergo a degradation model to simulate the information distribution of real-world images.

Image degradation models are widely applied in tasks such as image super-resolution reconstruction, image dehazing, and deblurring [25,26,27]. Infrared dim and small target images in real environments suffer from complex degradation mechanisms. If internal camera noise degradation is neglected, the degradation of moving target images can be divided into two cases: static background and dynamic background. Detecting static dim small targets is relatively easier, with the most direct influencing factor being noise degradation. Degradations such as random Gaussian blur, motion blur, and random scaling formed by changes in both targets and backgrounds present greater detection challenges.

Currently, isotropic Gaussian blur and anisotropic Gaussian blur are two commonly used image degradation methods. Since the motion direction of infrared sensors during real-world image acquisition is randomly variable, we assume that the images are affected by random anisotropic Gaussian blur. The causes of motion blur in infrared dim small targets can be attributed to three factors: (1) Imaging system shake: during imaging, slight shaking or movement of the camera causes blur in all targets in the image. (2) Target motion: movement of the target during imaging leads to changes in its position during the exposure time, leaving motion trails in the image. (3) Long exposure time: under low-light conditions with long exposure times, any slight movement can cause significant blurring effects [28].

Extensive research shows that image problems caused by multi-scale and random imaging distances can be equivalently treated as target random scale degradation due to resolution [29,30]. In addition, noise degradation, random target rotation, and radiation intensity degradation should also be considered as important imaging degradation modes [31,32].

## 3. Methods

In this section, we first present the TOA degradation model in Section 3.1. Subsequently, an online grid-based high-order constrained pseudo-sample generation method is designed in Section 3.2, where the formulations and detailed parameter settings of three constraint conditions are provided. Finally, a self-supervised training framework is constructed, including pseudo-label specification, training strategy, and loss function in Section 3.3.

### 3.1. TOA Degradation Model

**Figure 2:**
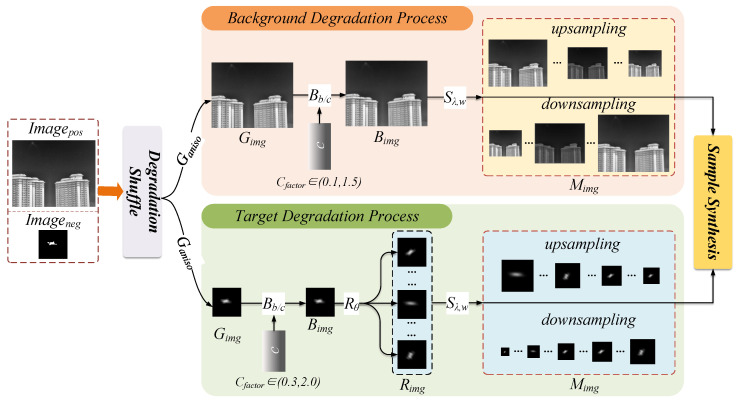
TOA image degradation model architecture.

Infrared dim and small target images in real-world environments undergo complex physical degradation during formation, exhibiting significant signal-to-noise ratio reduction, edge blurring, and radiation distortion. To systematically simulate this process, this paper proposes the Target-Oriented Adaptation (TOA) degradation model, whose overall architecture is shown in Figure 2. The core concept of the TOA model is to decouple the overall degradation process into a background degradation path and a target degradation path, thereby separately characterizing the texture evolution of background clutter and the optical attenuation characteristics of dim small targets.

The TOA model constructs a degradation function library comprising operations such as anisotropic blur, multi-scale sampling, and radiation intensity modulation. On this basis, a Degradation Shuffle mechanism is proposed, which achieves flexible control and diversified generation of the degradation process by randomly combining different degradation factors through information entropy optimization methods, enhancing the authenticity and coverage of synthetic samples.

In natural imaging processes, blurring caused by camera motion typically occurs first, while degradation in target radiation intensity and scale further develops based on the blurred images. This is because adding optical degradation factors to blurred images will further reduce the saliency of targets. Scale degradation usually refers to simulating distance changes or target volume variations. Applying scale changes last better aligns with the characteristics of long-distance, low-radiation targets in the imaging process, and can reduce the loss of target details after blur and brightness processing, preserving important feature information to facilitate subsequent pseudo-sample generation and target detection tasks.

Next, we will detail each degradation factor.

#### 3.1.1. Blur Degradation

During the infrared imaging process, influenced by the relative motion between the camera and the target, dim small targets and background information in real scenes often exhibit significant directional blur degradation characteristics. To simulate this physical process, this paper employs an anisotropic Gaussian blur model [33] to achieve directionally sensitive control of the blur effect.

A differentiated blur strategy is designed according to the degradation characteristics of background and target regions. Background regions typically contain rich textures and high-frequency clutter; therefore, larger random anisotropic Gaussian kernels with a wider standard deviation range are used to effectively smooth details and simulate background degradation in practical imaging. Target regions, especially dim small targets, are prone to loss due to excessive blur; hence, smaller Gaussian kernels with limited standard deviation are applied to maintain degradation rationality while avoiding loss of target saliency.

The mathematical formulation of the anisotropic Gaussian blur is as follows:

For the input image $I_{img}$, its blurred result $G_{img}$ is generated by a two-dimensional convolution operation:

(1) $$G_{img}=I_{img}*G\left(k_{w},k_{h},\sigma_{x},\sigma_{y}\right)$$

where ∗ denotes the convolution operation; $G(k_{w},k_{h},\sigma_{x},\sigma_{y})$ represents the anisotropic Gaussian kernel function, which is defined on a grid of size $k_{w}\times k_{h}$ with its center at (0,0). For any coordinate (x,y) on this grid, where $x\in \left[-\left(k_{w}/2\right),\left(k_{w}/2\right)\right],y\in \left[-\left(k_{h}/2\right),\left(k_{h}/2\right)\right]$, the value of the kernel function is defined as:

(2) $$G\left(x,y,\sigma_{x},\sigma_{y}\right)=\frac{1}{2\pi \sigma_{x}\sigma_{y}}exp\left(-\frac{1}{2}\left.\left(\left.\frac{x^{2}}{\sigma_{x}^{2}}+\frac{y^{2}}{\sigma_{y}^{2}}\right)\right.\right)\right.$$

where $k_{w}$ and $k_{h}$ denote the width and height of the Gaussian kernel, respectively; $\sigma_{x}$ and $\sigma_{y}$ represent the standard deviations in the two orthogonal directions, controlling the degree and anisotropy of the blur.

#### 3.1.2. Radiation Intensity Degradation

In real infrared imaging scenarios, non-uniform radiation intensity degradation often occurs in both background and target regions due to variations in environmental illumination, manifesting as overall grayscale distortion or local contrast reduction [34]. Background areas are susceptible to influences such as low light or overexposure, leading to global shifts or compression in their grayscale distribution. In contrast, dim-small targets exhibit more pronounced brightness fluctuations and contrast attenuation caused by imaging distance, orientation, and environmental stray light. Such degradations are typically more prominent in the grayscale domain.

To accurately simulate this complex radiation distortion process, we introduce a linear radiation response model that jointly accounts for both brightness and contrast degradation. This model learns the statistical distribution characteristics of radiation intensity in real environments by randomly sampling a gain factor and an offset factor. The degradation function is formulated as:

(3) $$B_{img}=I_{img}\times g_{factor}+o_{factor}$$

where $B_{img}$ denotes the degraded image; $I_{img}$ denotes the input image; $g_{factor}\in \left(g_{min},g_{max}\right)$ is a random gain factor simulating the contrast degradation process, and $o_{factor}\in \left(o_{min},o_{max}\right)$ is a random offset factor simulating the overall brightness degradation.

#### 3.1.3. Scale Degradation

In the generation of pseudo-samples for infrared dim and small targets, scale degradation is a key aspect for simulating variability in real imaging conditions. Scale degradation of the background primarily arises from differences in camera models, resolution configurations, and imaging distances. In contrast, scale degradation of targets is significantly influenced by both imaging distance and changes in the target’s own posture. Therefore, modeling target scale requires comprehensive consideration of both random size degradation and random posture degradation [35].

To address this, we construct a scale degradation function, expressed mathematically as:

(4) $$M_{img}=R_{\theta }\left(S_{\lambda ,w}\left(I_{img}\right)\right)$$

where $S_{\lambda ,w}\left(\cdot \right)$ denotes a stochastic resampling function. The scaling factor λ is randomly selected from the set {2,3,4}, indicating that the width and height of the input image $I_{img}$ are rescaled to λ times (upsampling) or 1/λ times (downsampling) of the original dimensions. The sampling method *w* is randomly chosen from {nearest-neighbor, bilinear, bicubic} to simulate the reconstruction differences introduced by various interpolation algorithms. $R_{\theta }\left(\cdot \right)$ represents a random rotation transformation, with the rotation angle θ uniformly generated within the range of $\left(-60^{\circ },60^{\circ }\right)$, used to simulate the apparent target variations caused by relative pose changes. It is important to note that the scale degradation for background samples applies only $S_{\lambda ,w}\left(\cdot \right)$, without incorporating the pose rotation transformation.

The selection of the λ range is based on considerations of the physical constraints of the imaging system. As mentioned in Section 3.2.2, the input images are standardized to a resolution of 512×512. Choosing {2,3,4} ensures that the resolution of the generated samples remains within a reasonable interval of approximately 170×170 to 2048×2048. This range not only covers mid-to-long-range scale variations but also avoids producing invalid samples with excessively low or high resolutions, thereby ensuring the physical plausibility of the simulated data.

### 3.2. Pseudo-Sample Generation

As described in Section 2.2, directly using original background and target samples cannot effectively drive the network to learn the distribution of real image information. Moreover, images processed through degradation still require strict constraints during pseudo-sample synthesis to accurately simulate the actual imaging mechanism.

**Figure 3:**
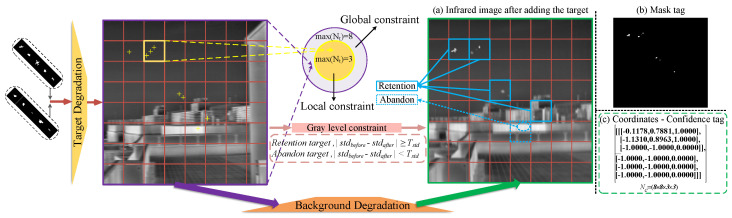
The framework of the high-order constraint pseudo-sample generation algorithm proposed in this paper.

To address this, this paper proposes a grid-based high-order constrained pseudo-sample generation method. This approach collaboratively generates high-fidelity pseudo-sample data with authentic image information through global target constraints, local target constraints, and local grayscale-level constraints. The overall framework of the method is shown in Figure 3, with the algorithm pseudo-code provided in Algorithm 1. The method does not restrict the number of generated targets and allows flexible setting of the target quantity range to adapt to different task requirements.
**Algorithm 1** Pseudo-code of Pseudo-Sample Generation**Require:** $I_{b}$: background image; $I_{t}$: target images; *A*: anchor size for grid partitioning; $M_{o}$: maximum number of objects per image; $T_{a}$: maximum number of targets per anchor **Ensure:** $I_{g}$: generation image; $I_{m}$: mask, *L*: $Label_{cc}$  1:Initialize: V(I): variance compute; $R_{pos}$($U,w,h,w_{s},h_{s}$): Function to generate a random position (x,y); τ: threshold interval; L←0; U←∅  2:$I_{b-d}\leftarrow \mathrm{TOA}\left(I_{b}\right)$  3:Sample $N_{o}∼U\left(1,M_{o}\right)$  4:**for** i=1 **to** $N_{o}$ **do**  5: $I_{t-d}\leftarrow \mathrm{TOA}\left(I_{t}\right)$  6: $\left(h_{s},w_{s}\right)\leftarrow \mathrm{size}\;(I_{t-d}$); $\left(h,w\right)\leftarrow \mathrm{size}\;(I_{b-d}$)  7: $\left(x,y\right)\leftarrow R_{pos}\left(U,w,h,w_{s},h_{s}\right)$  8: U←U∪{(x,y)}  9: Compute $\sigma_{before}\leftarrow V\left(I_{b-d}\left[x:x+w_{s},y:y+h_{s}\right]\right)$10: Blend $I_{t-d}$ into $I_{b-d}\left[x:x+w_{s},y:y+h_{s}\right]$11: Compute $\sigma_{after}\leftarrow V\left(I_{b-d}\left[x:x+w_{s},y:y+h_{s}\right]\right)$12: **if** $∥\sigma_{before}-\sigma_{after}∥\in \tau$ **then**13:  $I_{g}\leftarrow I_{b-d}$14:  $I_{m}\left[x:x+w_{s},y:y+h_{s}\right]\leftarrow I_{m}$15:  $\left(c_{x},c_{y}\right)\leftarrow \left(x+w_{s}/2,y+h_{s}/2\right)$16:  $\left(a_{x},a_{y}\right)\leftarrow \left(\left\lfloor c_{x}/A\right\rfloor ,\left\lfloor c_{y}/A\right\rfloor \right)$17:  $\Delta_{x}\leftarrow \frac{c_{x}\%A}{A}-0.5;\Delta_{y}\leftarrow \frac{c_{y}\%A}{A}-0.5$18:  Update $L\left[a_{x},a_{y},\mathrm{next}\right]\leftarrow \left(\Delta_{x},\Delta_{y},1\right)$19: **else**20:  Delete (x,y)21: **end if**22:**end for**23:Normalize $I_{g}$ and apply T if defined24:Return $I_{g},I_{m},L$

#### 3.2.1. Global Target Constraint

Global constraints are primarily applied to both target quantity and spatial distribution. First, the total number of targets *N* added to the entire image is constrained to satisfy $N\in [0,N_{\max}]$, ensuring the reasonableness of target quantity and consistency across samples. Second, for an image size of H×W, target positions $\left(x_{i},y_{i}\right)$ (where *i* denotes the target index) are set according to the content distribution characteristics of the images in the dataset. For images with simple and uniform content, targets can be randomly added across the entire image, i.e., $0\leq x_{i}\leq W-1$, $0\leq y_{i}\leq H-1$. For complex scene images containing multiple elements such as sky, buildings, trees, and pedestrians, targets should be preferentially added to the upper region of the image, with a minority distributed in other areas.

This constraint can be formally expressed as:

(5) $$\left\{\begin{array}{c} \begin{array}{c} \underset{i=1}{\sum^{N}}I\left(y_{i}\leq \frac{H}{\varepsilon }\right)\geq \alpha N\\ \underset{i=1}{\sum^{N}}I\left(y_{i}>\frac{H}{\varepsilon }\right)\leq \left(1-\alpha \right)N \end{array} \end{array}\right.,\;\left(\alpha >\varepsilon \right)$$

where *I* is the indicator function that equals 1 when the condition is satisfied and 0 otherwise; α is the proportion parameter, and ε controls the division ratio of the upper region.

#### 3.2.2. Local Target Constraint

To enhance the local authenticity of generated samples, this paper further introduces grid-based local constraints. First, the image is uniformly scaled to a resolution of 512×512 and divided into 8×8 image blocks $B_{ij}$ each with a size of 64×64:

(6) $$B_{ij}=I\left[64i:64\left(i+1\right)-1,64j:64\left(j+1\right)-1\right],i,j=0,1,2,\ldots ,7$$

Subsequently, the number of targets $N_{i,j}$ in each image block $B_{ij}$ is constrained to $N\in [0,N_{\max}]$. This local target constraint effectively avoids the issue of uneven target distribution in local regions and eliminates the occurrence of “pseudo-targets”.

#### 3.2.3. Local Grayscale Constraint

After the degradation processing described in Section 3.1, the visibility of some target samples is significantly reduced. Moreover, global and local positional constraints alone cannot guarantee the compatibility of radiation characteristics between targets and backgrounds. If a target is placed in an area with highly similar radiation features, it should be excluded. To address this, the grayscale values of image block $B_{ij}$ before and after target generation are defined as $I_{ijk}$ and $I_{ijk}^{\prime }$ (where *k* is the pixel index), and a tolerable local standard deviation change threshold $\Delta \sigma_{th}$ is set to evaluate the rationality of target placement:

(7) $$\sigma_{pre}^{ij}=\sqrt{\frac{1}{n}\underset{k=1}{\sum^{n}}\;{\left(I_{ijk}-\mu_{ij}\right)}^{2}},\sigma_{post}^{ij}=\sqrt{\frac{1}{n}\underset{k=1}{\sum^{n}}\;{\left(I_{ijk}^{\prime }-\mu_{ij}^{\prime }\right)}^{2}}$$

where *n* denotes the total number of pixels in the image patch $B_{ij}$; $\sigma_{pre}^{ij}$ and $\sigma_{post}^{ij}$ are the standard deviations within the block before and after adding the target, respectively; $\mu_{ij}$ and $\mu_{ij}^{\prime }$ are the average grayscale values of the corresponding block. If the condition $\Delta \sigma_{ij}=∥\sigma_{post}^{ij}-\sigma_{pre}^{ij}∥\in \Delta \sigma_{th}$ is satisfied, the target placement at this location is considered radiometrically reasonable and consistent with real imaging characteristics.

### 3.3. Self-Supervised Learning Framework

**Figure 4:**
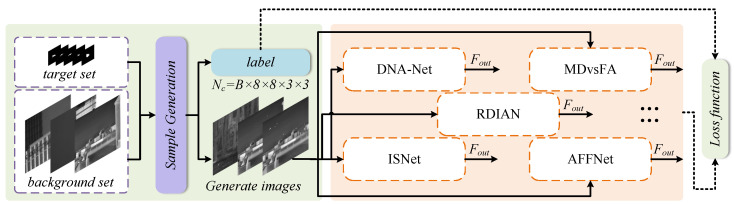
The self-supervised learning training framework proposed in our paper.

This paper constructs a complete self-supervised learning framework consisting of three components: pseudo-sample generation, a neural network library, and an adaptive training strategy. The overall structure is shown in Figure 4. The framework takes the degraded samples and corresponding labels generated earlier as input, optimizes the detection model in an end-to-end manner, and finally validates its generalization capability on real-world data.

#### 3.3.1. Pseudo-Labels

Traditional grid labels rigidly assign targets to specific grid cells. This discrete assignment method tends to introduce annotation noise when targets are near grid boundaries or partially occluded, leading to unstable training and limited localization accuracy.

Unlike the widely used fixed grid labels, the pseudo-labels generated in this study are target position-confidence array labels of size Batch×8×8×3×3. Through soft assignment and continuous supervision of target positions, they more finely reflect the spatial distribution of targets. This approach is particularly beneficial for improving the detection performance of small and occluded targets.

#### 3.3.2. Training Strategy

The training process adopts an end-to-end supervised approach. Using the pseudo-samples and their corresponding pseudo-labels generated in Section 3.2 as training data, parameter optimization is achieved by minimizing the difference between the network output and the pseudo-labels. The loss function employs the position-confidence loss function $L_{cc}$ defined in Section 3.3.3.

#### 3.3.3. Loss Function

To compute the loss with pseudo-labels, we design a position-confidence loss function ($L_{cc}$). The $L_{cc}$ introduces a weighted combination of position loss ($L_{cd}$) and confidence loss ($L_{cf}$), which ensures accurate judgment of target existence while enhancing the fine-grained prediction capability of target locations. The kernel of both $L_{cf}$ and $L_{cd}$ uses the $L_{2}$-norm [36].

Since The output of the neural network is a five-dimensional array *P* of size Batch×8×8×3×3. The last dimension of *P* and the pseudo-label five-dimensional array *T* contains the target’s position offset and confidence information, respectively. Specifically, P[…,0:2] or T[…,0:2] represents the predicted or labeled two-dimensional position coordinates (x,y), while P[…,2] or T[…,2] indicates the predicted or labeled target confidence.

Therefore, $L_{cd}$ can be expressed as:

(8) $$\begin{array}{cc} L_{cd} & =\sum_{i,j,k,l}{\left(M_{cood}\cdot P_{pos}\left[i,j,k,l\right]-M_{cood}\cdot T_{pos}\left[i,j,k,l\right]\right)}^{2} \end{array}$$

where $P_{pos}\left[i,j,k,l\right]$ denotes the two-dimensional coordinates (x,y) predicted by the network, corresponding to the original array P[i,j,k,l,0:2]; $T_{pos}\left[i,j,k,l\right]$ represents the two-dimensional coordinates of the pseudo-label at the corresponding position, i.e., the original array T[i,j,k,l,0:2]; and $M_{cood}$ is a mask (also a five-dimensional array) generated based on the pseudo-label confidence, ensuring that only positions with non-zero confidence participate in the loss calculation.

The $L_{cf}$ can be expressed as:

(9) $$L_{cf}=\sum_{i,j,k,l}(P_{conf}\left[i,j,k,l\right]-T_{conf}\left[i,j,k,l\right]{)}^{2}$$

where $P_{conf}\left[i,j,k,l,2\right]$ denotes the target confidence predicted by the network, i.e., the original array P[i,j,k,l,2]; and $T_{conf}\left[i,j,k,l,2\right]$ represents the target confidence of the pseudo-label, i.e., the original array P[i,j,k,l,2].

Finally, the $L_{cc}$ loss function is defined as:

(10) $$L_{cc}=\lambda_{1}\cdot L_{cd}+\lambda_{2}\cdot L_{cf}$$

where $\lambda_{1}$ and $\lambda_{2}$ are balance factors for the position and confidence losses, respectively. When dealing with datasets containing a larger number of target categories or greater scale variations, the training process can be optimized by adjusting $\lambda_{1}$ and $\lambda_{2}$.

## 4. Experimental

### 4.1. Datasets Settings

**Figure 5:**
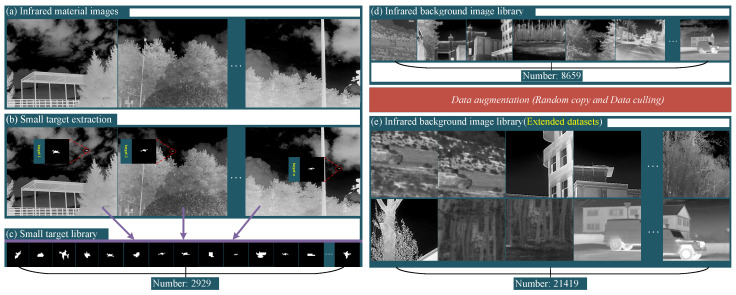
Description of the simulated dataset acquisition process. (**a**) Original image library for acquiring small target binary labels. (**b**) Display of acquired small target binary images. (**c**) Acquired a library of 2929 small target label images with a size of 30×30 pixels. (**d**) Acquired library of 8659 infrared background images with a size of 512×512 pixels, to which small targets need to be added. (**e**) Infrared background image library containing 21,419 images after data augmentation.

**A. For network training.** Background samples are shown in Figure 5e. We selected 6000 images from public datasets including FLIR [37], TNO [38], and MSRS [39] as background samples. Additionally, we constructed an infrared image acquisition platform to collect 2659 custom background samples as Supplementary Data. Finally, the background sample images were expanded to 21,419 through data augmentation techniques. Target samples are shown in Figure 5c. We first acquired three types of infrared drone small target images. Then, we designed a small target morphology detection algorithm to obtain 1500 real target samples with size of 30×30 pixels. Furthermore, we generated 1429 simulated target samples with random morphologies through simulation. Thus, the total number of target sample images reached 2929.

**B. For network testing.** We used public real datasets NUAA-SIRST (ACM) [12] and IRDST-real (RDIAN) [7] for evaluation. Since the labels of these two datasets lack the confidence dimension, we extended them with a confidence dimension to match the output format of our model.

### 4.2. Network Training Details

All software components were implemented using the open-source PyTorch 2.4.0 framework. Hardware training, testing, model tuning, and subsequent program modularization were performed on a computer equipped with an NVIDIA GeForce RTX 4090 GPU. In the loss function $L_{cc}$ used in this paper, the balance factors for the position loss and confidence loss were set to $\lambda_{1}$ = 0.2 and $\lambda_{2}$ = 0.8, respectively. During network training, the learning rate was set to 0.01, and network optimization was performed using SGD gradient descent with a batch size of 32. The network achieved stable convergence after 200 epochs.

### 4.3. Evaluation Metrics

To comprehensively evaluate the performance of the proposed method, a quantitative evaluation system is established from two dimensions: pseudo-sample generation quality and target detection performance.

#### 4.3.1. Pseudo-Sample Quality Metrics

To assess the authenticity of the pseudo-samples, the Signal-to-Clutter Ratio (SCR) is adopted to evaluate the difficulty of the target detection task. The SCR is defined as:

(11) $$SCR=\frac{\mu_{t}-\mu_{b}}{\sigma_{b}}$$

where $\mu_{t}$ represents the grayscale intensity of the target region, while $\mu_{b}$ and $\sigma_{b}$ denote the mean and standard deviation of the background region, respectively.

Information Entropy (IE) is used to describe the texture complexity and information richness of the image. The IE is defined as:

(12) $$IE=-\sum_{i=0}^{255}p_{i}\cdot {\log_{2}}^{p_{i}}\;$$

where $p_{i}$ indicates the probability of the corresponding grayscale intensity.

#### 4.3.2. Detection Metrics

To evaluate the target localization accuracy of the proposed method, we design a Position Deviation (Pod) metric, which measures the average Euclidean distance deviation between the predicted target positions and the ground truth positions, directly reflecting the localization precision. The Pod is formulated as:

(13) $$Pod=\frac{1}{N_{label}}\underset{i=1}{\sum^{N_{label}}}\;{\left\|{\left(x,y\right)}_{output,i}-{\left(x,y\right)}_{label,i}\right\|}_{2}$$

where $N_{label}$ denotes the number of real targets in the pseudo-labels; ${\left(x,y\right)}_{output,i}$ and ${\left(x,y\right)}_{label,i}$ represent the predicted coordinates and the ground truth coordinates in the pseudo-labels of the *i*-th target, respectively. This metric measures the average pixel distance, with a value range of [0,+∞) and units of pixels. A smaller value indicates higher localization accuracy.

Furthermore, we employ the F1−measure (F1), precision rate (Prec), false alarm rate ($F_{a}$), and Intersection over Union (IoU) [40] metrics to comprehensively evaluate the performance of the proposed method. These metrics (except for $F_{a}$) are proportional values with a range of [0, 1] and are expressed in percentages (%). Larger values indicate better performance.

The F1 represents the harmonic mean of precision and recall, providing a comprehensive assessment of the detection performance. It is calculated as:

(14) $$F1=\frac{2\times Prec\times Rec}{Prec+Rec}$$

where Prec and Rec denote the precision rate and recall rate, respectively.

The precision rate (Prec) indicates the proportion of true positive targets among all detections predicted by the model as targets. It is calculated as:

(15) $$Prec=\frac{TP}{TP+FP}$$

where TP represents the number of true positive targets correctly detected by the model, and FP denotes the number of false positive targets incorrectly detected by the model.

The false alarm rate ($F_{a}$) quantifies the number of false targets per unit area or unit quantity. In this paper, it is defined as the number of false alarms per million pixels. The formula is:

(16) $$F_{a}=\frac{FP}{N_{total}}\times 10^{6}$$

where $N_{total}$ represents the normalized total number of reference pixels. The value range of $F_{a}$ is [0,+∞), with units of $10^{-6}$. A smaller value indicates better performance.

The Intersection over Union (IoU) measures the overlap between the predicted target bounding box and the corresponding ground truth bounding box. It is calculated as:

(17) $$IoU=\frac{A_{P}⋂A_{T}}{A_{P}⋃A_{T}}$$

where $A_{P}$ represents the area of the predicted box, and $A_{T}$ represents the area of the ground truth box.

### 4.4. Comparation

To evaluate the training foundation provided by pseudo-samples for self-supervised learning, this study conducts comparative experiments with datasets generated by five classical pseudo-sample synthesis methods. The compared datasets include: the NUDT-SIRST dataset proposed by DNA-Net [13], the NUST-SIRST dataset proposed by MDvsFA [41], the IRDST-simulation dataset proposed by RDIAN [7], the IRSTD-1K dataset proposed by ISNet [42], and the SIRST-5K dataset proposed by AFFNet [20].

#### 4.4.1. Pseudo-Sample Quality Comparison

**Figure 6:**
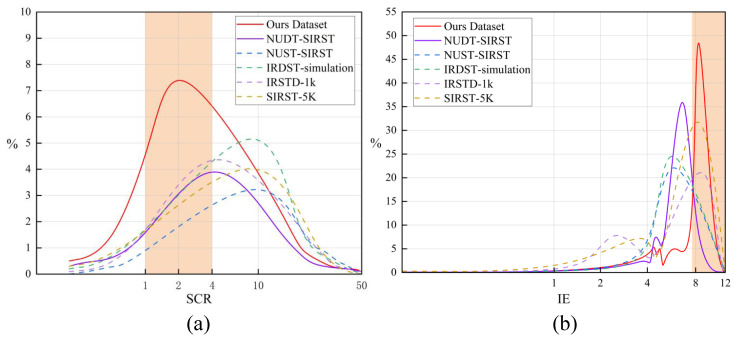
Distribution of the proposed pseudo-sample quality in terms of SCR and IE. (**a**) The x-axis represents the SCR range (0–50), where lower values indicate more challenging detection targets. The y-axis shows the proportion of samples under different SCR values relative to the total dataset. (**b**) The x-axis represents image complexity and information content, while the y-axis indicates the proportion of samples under different IE values relative to the total dataset. The orange area represents the distribution of high-quality pseudo-samples. The more data points in this area, the better the quality. The pseudo-samples obtained in this study exhibit higher detection complexity, richer information content, and greater structural authenticity.

This section presents a quantitative comparison between the generated pseudo-samples and the five aforementioned simulated datasets using the SCR and IE metrics. The results are shown in Figure 6a,b. It can be observed that the pseudo-samples proposed in this paper outperform existing simulated datasets in terms of image complexity and realism.

#### 4.4.2. Comparison of Detection Performance

**A. Quantitative comparison.** To comprehensively evaluate the effectiveness and generalization ability of the pseudo-samples generated in this paper, we conduct cross-training experiments using five baseline detection networks (DNA-Net, MDvsFA, RDIAN, ISNet, SIRST-5K) with six simulated datasets (including the pseudo-sample dataset generated in this paper and five baseline simulated datasets). The evaluation is performed on two real infrared dim and small target datasets (NUAA-SIRST and IRDST-real). The quantitative results are presented in Table 1 and Table 2.

As shown in Table 1, the RDIAN network trained with the pseudo-samples from this paper achieves four optimal performance metrics on the real NUAA-SIRST dataset, significantly outperforming results trained with other simulated data. It is worth noting that when trained using the NUST-SIRST simulated dataset, which has high homology with the real test set, RDIAN only performs well on some metrics. This indicates that network performance is still affected by the distribution similarity between the training and test sets, reflecting the limitations of traditional simulation data in cross-scene generalization.

The results in Table 2 further demonstrate that models trained with the proposed method maintain stable advantages on the IRDST-real dataset, particularly excelling in target localization accuracy. The RDIAN baseline model achieves two optimal values and one sub-optimal value. In contrast, although the model trained with the homologous IRDST-simulation data achieves some relatively good results when testing on IRDST-real, its performance significantly depends on the scene homology between the training and test sets. This again confirms the limitations of such methods in cross-scene generalization capability.

**Figure 7:**
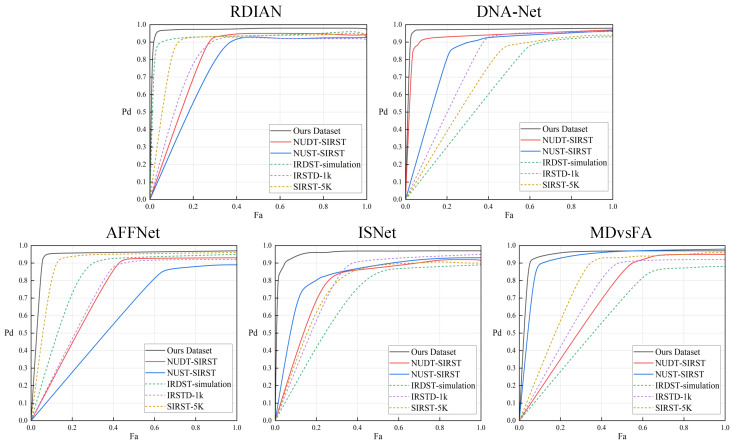
ROC curve evaluation of five networks trained on six simulated datasets, respectively.

Figure 7 shows the ROC curves of five networks trained on six types of simulated data. It can be observed that regardless of the base network architecture, training with the pseudo-samples generated in this paper enables the models to converge faster to better performance regions, and the area under the curve (AUC) is generally higher than results trained with other simulated data. This indicates that the proposed pseudo-samples better approximate real infrared imaging characteristics in terms of feature distribution, noise structure, and target-background interaction relationships, thereby supporting stable detection performance in unknown real-world scenarios.

**Figure 8:**
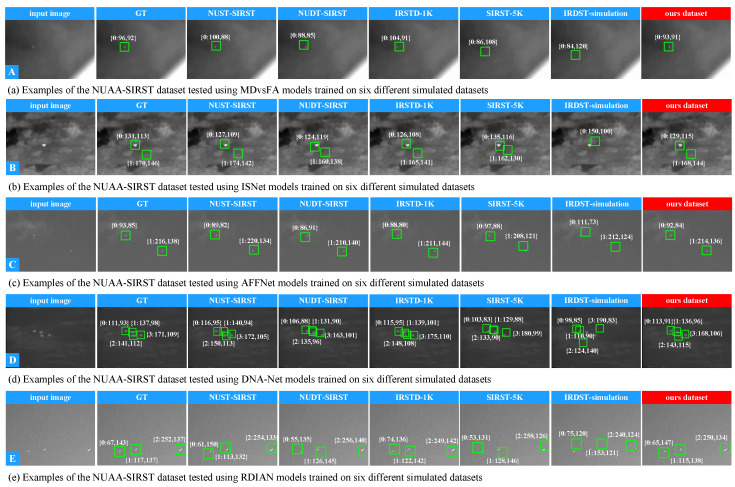
Qualitative visualization results on the real dataset NUAA-SIRST. Each row of images corresponds to the centroid localization results of five baseline networks: the first column shows the original infrared image, the second column shows the ground truth centroid annotation, and the third to eighth columns display the detection results of models trained on the six simulated datasets. It can be clearly observed that the self-supervised learning method proposed in this paper outperforms the comparative methods in terms of false alarm rate, detection rate, and localization accuracy.

**Figure 9:**
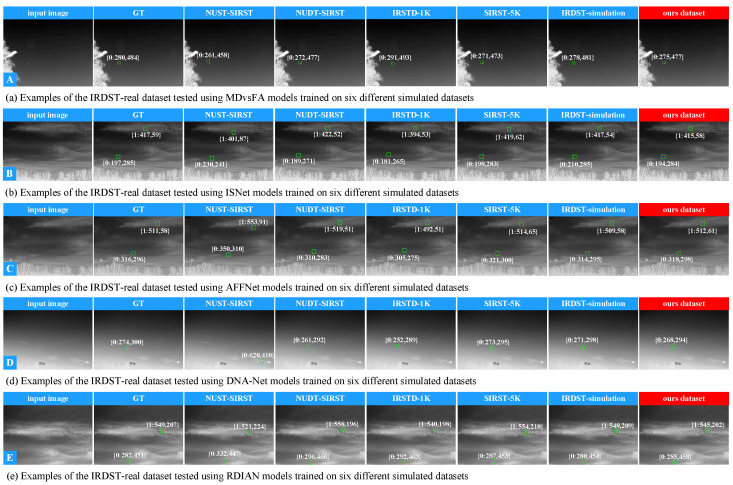
Qualitative visualization results on the real dataset IRDST-real. Each row of images represents the centroid visualization results of five baseline networks. The first column displays the original image; the second column shows the ground truth centroid visualization; and the third to eighth columns present the centroid visualizations of detection results from models trained on the six simulated datasets. It can be clearly seen that the self-supervised learning method proposed in this paper achieves a lower false alarm rate, a higher detection rate, and superior target localization accuracy.

**B. Qualitative comparison.** To validate the effectiveness of the proposed method, we randomly selected 10 representative single-target and multi-target infrared images from the NUAA-SIRST and IRDST-real datasets for testing. The experimental results are shown in Figure 8 and Figure 9. Since the maximum number of targets in the IRDST-real dataset is two, the multi-target test only includes dual-target scenarios. To further analyze the detection accuracy of different methods, we simultaneously visualized the centroid localization results of each method.

Taking the single-target scenario as an example (Figure 8a), the centroid localization deviation of the proposed method is only 4 pixels, while the deviations of other methods are 8, 9, 15, 26, and 40 pixels, respectively. In the multi-target scenario (Figure 8e), the deviation of the proposed method is 14 pixels, while the deviations of other methods are 28, 32, 44, 63, and 105 pixels, respectively. The following conclusions can be drawn: (1) The adaptability of detection models to single-target and multi-target tasks differs, with single-target detection being significantly easier; (2) The catastrophic forgetting problem still exists in infrared dim and small target detection tasks and has a significant impact on model performance. The superior performance of the proposed method mainly benefits from the TOA simulation mechanism, which covers diverse imaging conditions in real environments, generates infrared samples with varying morphologies, sizes, visibility levels, and background complexities, and introduces high-order degradation constraints during pseudo-sample generation, thereby effectively enhancing the generalization capability of the model.

Figure 9 presents the qualitative comparison results on the IRDST-real dataset. Combined with the quantitative data in Table 3, it can be observed that the proposed method achieves the lowest total positional deviation among all models, with a value of 58 pixels. In comparison, the total deviations of models trained on NUST-SIRST, NUDT-SIRST, IRSTD-1K, SIRST-5K, and IRDST-simulation are 844, 153, 216, 76, and 56 pixels, respectively. This result further verifies that the proposed method exhibits superior localization accuracy and stability in real-world scenarios.

### 4.5. Ablation Study

This section conducts detailed ablation studies on the TOA degradation model, the high-order constraint algorithm, and the loss balance factor to validate the effectiveness of the proposed pseudo-sample generation method and the positional deviation rate. All experiments are performed under identical training settings to ensure a fair comparison.

#### 4.5.1. Ablation Study on TOA

**Figure 10:**
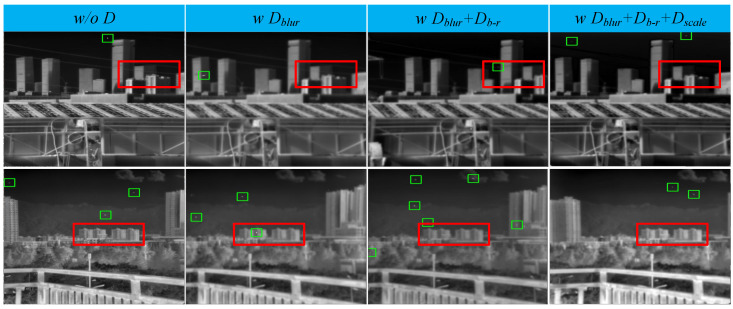
Pseudo-sample images generated by four different degradation schemes (magnification recommended for detailed observation). From left to right: No degradation, $D_{blur}$, $D_{blur}$ + $D_{b-r}$, and $D_{blur}$ + $D_{b-r}$ + $D_{scale}$.

This subsection examines the impact of pseudo-samples generated under different degradation configurations on the detection model: no degradation (Scheme A), sequentially adding blur degradation $D_{blur}$ (Scheme B), radiation intensity degradation $D_{b-c}$ (Scheme C), and scale degradation $D_{scale}$ (Scheme D). The results are shown in Table 4 and Figure 10. To augment the samples, random cropping has been applied in each scheme.

As shown in Table 4, the detection model trained on pseudo-samples without any degradation experiences a sharp decline in performance on the real dataset. In contrast, consistent performance improvements are observed as blur degradation $D_{blur}$, radiation intensity degradation $D_{b-c}$, and scale degradation $D_{scale}$ are incrementally incorporated. This occurs because models trained on data generated without degradation fail to simulate the degradation mechanisms present in real images, leading to poor generalization. It is worth noting that in tests on IRDST-real, which contains images with extremely small target sizes, the model trained with the addition of $D_{scale}$ shows a significant performance boost.

Next, we perform detailed ablation experiments for each type of degradation. The default configuration includes all three degradation types, with only one degradation type’s configuration altered at a time.

**A. Blur Degradation ($D_{blur}$).** To validate the impact of blur degradation modeling on detection performance, we compare traditional motion blur degradation (Scheme A) with the proposed anisotropic Gaussian blur degradation method (Scheme B). Using the NUAA-SIRST and IRDST-real real datasets described in Section 4.1 for testing, we evaluate the effectiveness of different blur modeling approaches within the self-supervised learning framework. The results are shown in Table 5.

Table 5 demonstrates that Scheme B (anisotropic Gaussian blur) significantly outperforms Scheme A (traditional motion blur) across all evaluation metrics. Specifically, on the NUAA-SIRST dataset, the Pod of Scheme B increases from 0.697 to 0.736, the Prec improves from 98.013 to 98.841, and the $F_{a}$ decreases from 2.994 to 2.614. A consistent trend is observed on the IRDST-real dataset, where Scheme B maintains a high detection rate while achieving notably better localization deviation and false alarm rates compared to Scheme A. These results fully demonstrate that the anisotropic Gaussian blur degradation method adopted in this paper can more accurately simulate the direction-sensitive blur effects caused by target-sensor relative motion and atmospheric turbulence in real imaging processes.

**B. Radiation Intensity Degradation ($D_{b-c}$).** We retrained two detection models using the pseudo-samples generated in this paper for comparison: radiation intensity degradation applied only to targets (Scheme A), radiation intensity degradation applied only to backgrounds (Scheme B), and complete radiation intensity degradation applied to both targets and backgrounds (Scheme C). The radiation intensity degradation factor $c_{factor}$ in this section is set according to Equation (4). The networks are also tested on two real datasets to validate the impact of radiation intensity degradation on self-supervised learning. The results are shown in Table 6.

As shown in Table 6, the complete radiation intensity degradation (Scheme C) achieves three optimal values on both real test datasets, verifying that both infrared backgrounds and targets undergo radiation intensity degradation in the real world. The proposed complete radiation intensity degradation effectively enhances target detection performance under random brightness distributions. The schemes with degradation applied only to targets (Scheme A) or only to backgrounds (Scheme B) each achieve three sub-optimal values on the two test sets. This indicates that the NUAA-SIRST dataset exhibits relatively severe background degradation, while target information is more prominent compared to the IRDST-real dataset. Conversely, the IRDST-real dataset suffers from significant target degradation, while background information is clearer compared to the NUAA-SIRST dataset.

**C. Scale Degradation ($D_{scale}$).** In this section, we retrain two detection models for comparison: scale degradation applied only to the background (Scheme A), scale degradation applied only to the target (Scheme B), and complete scale degradation applied to both target and background (Scheme C). The sampling factor and rotation factor are set according to Equation (4). We validate the generalization ability of models trained on simulated data generated with the scale degradation mechanism on real data. The results are shown in Table 7.

As shown in Table 7, the complete scale degradation Scheme C achieves a total of six optimal values across the two real datasets. The Prec metric improves by 2.23 and 0.07 points compared to Scheme A, and by 1.69 and 1.10 points compared to Scheme B, respectively, in the two real datasets. This verifies the importance of scale degradation in enhancing dim and small target detection performance. Particularly in IRDST-real, scale degradation significantly alleviates the randomness in target sample scale and pose.

#### 4.5.2. Ablation Study on Pseudo-Sample Generation

**Figure 11:**
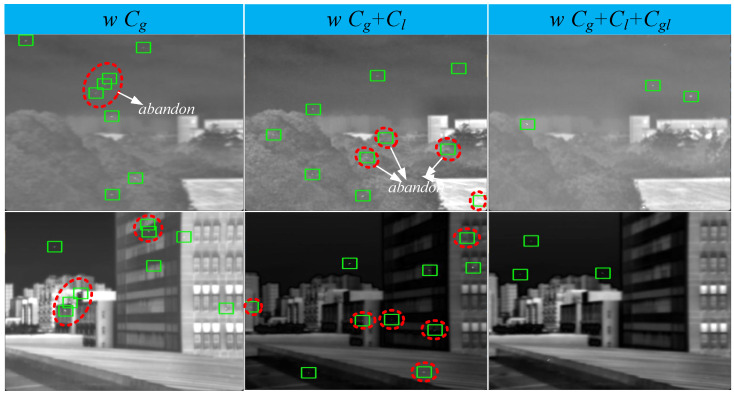
Pseudo-sample images generated by three different constraint schemes (magnification recommended for detailed observation). From left to right: $C_{g}$, $C_{g}$ + $C_{l}$ and $C_{g}$ + $C_{l}$ + $C_{gl}$.

In this section, we conduct a stepwise ablation experiment on the three constraint conditions for pseudo-sample generation to validate whether the hierarchical constraint strategy of the three constraint methods ($C_{g}$, $C_{l}$, and $C_{gl}$) in pseudo-sample generation has a significant impact on the trained detection models. We also train two detection models: one with only the global target constraint ($C_{g}$), one with the global target constraint plus the local target constraint ($C_{g}$ + $C_{l}$), and one with the complete set of global target constraint + local target constraint + local grayscale constraint ($C_{g}$ + $C_{l}$ + $C_{gl}$). The three schemes are tested on two real datasets, and the experimental results are shown in Table 8 and Figure 11.

As shown in Table 8, the complete constraint scheme $C_{g}$ + $C_{l}$ + $C_{gl}$ achieves a total of nine optimal values across the three evaluation metrics on the two real test datasets. The scheme $C_{g}$ + $C_{l}$ achieves seven sub-optimal values on the two real test datasets. Furthermore, it is evident that compared to the evaluation metrics in each ablation scheme for image degradation, the differences in evaluation metrics among the various ablation schemes for pseudo-sample generation show a gradually narrowing trend. This verifies that the proposed pseudo-sample generation constraint method effectively complements the detection model on the basis of image degradation.

As shown in Figure 11, to more subjectively observe whether the number, position, and global layout of pseudo-sample targets generated by the three constraint schemes are closer to the real data distribution, we visualize the results of the three constraint schemes. We use red closed curves to mark unreasonable targets generated by the incomplete pseudo-sample generation schemes. The addition of local constraints and local grayscale constraints results in generated targets with higher local quantity and global realism quality.

#### 4.5.3. Ablation Study on $\lambda_{1}$ and $\lambda_{2}$

To validate the impact of the position loss weight $\lambda_{1}$ and the confidence loss weight $\lambda_{2}$ on model performance, and to explore the rationality of the optimal weight combination (0.2, 0.8), this section designs an ablation experiment on the loss balance factors. The results are shown in Table 9.

The experiment first satisfies the condition $\lambda_{1}+\lambda_{2}=1$, then selects multiple different combinations of ($\lambda_{1}$, $\lambda_{2}$) = {(0.1, 0.9), (0.2, 0.8), (0.3, 0.7), (0.4, 0.6), (0.5, 0.5), (0.6, 0.4), (0.7, 0.3), (0.8, 0.2), (0.9, 0.1)}. All other hyperparameters (learning rate, batch size, network architecture, etc.) and training settings (dataset, number of iterations, etc.) are kept consistent.

As can be seen from Table 9, the configuration of the loss balance factors $\lambda_{1}$ and $\lambda_{2}$ has a significant non-linear impact on model performance. The optimal combination ($\lambda_{1}$ = 0.2, $\lambda_{2}$ = 0.8) demonstrates the best performance, with the model beginning to converge after only 23 epochs, which is 26% faster than the sub-optimal combination (0.3, 0.7). In-depth analysis reveals that when the ratio $\lambda_{1}$/$\lambda_{2}$ remains within the interval of 4.0 ± 0.5, the model performance shows a stable plateau. Performance drops sharply outside this range, validating the physical rationality of the confidence-dominated strategy, indicating that target existence judgment is the foundation for precise localization.

## 5. Discussion

### 5.1. Limitations Analysis

The proposed TOA degradation model effectively simulates the “dim, weak, and small” physical characteristics of infrared dim-small targets by integrating blur, radiation intensity, and scale degradations. However, this study has two main limitations. First, the designed degradation pipeline does not yet fully replicate all disturbance factors present in real-world environments, particularly in modeling complex background noise, which somewhat restricts the comprehensiveness of the synthetic data. Second, the degradation factors in our method are set globally based on the entire dataset, lacking adaptive adjustment according to the semantic content of individual images. This may lead to the loss of critical information in some samples under strong degradation.

To address these issues, future work will focus on the following directions: First, we will explore and integrate more sophisticated noise models and physical radiation transfer models to enhance the diversity of degradations. Second, we will investigate an adaptive degradation mechanism based on learnable information channels [43], enabling content-aware modulation of degradation factors to better preserve image fidelity. Third, we plan to incorporate continual learning paradigms [44] to improve feature generalization and mitigate catastrophic forgetting in cross-database scenarios.

### 5.2. Feasibility Analysis

The generalization capability of deep learning models in real-world scenarios is highly dependent on the distributional consistency between the training data and real-world data. However, acquiring large-scale, high-quality, and accurately annotated real datasets is often time-consuming, labor-intensive, and costly, which has become a critical bottleneck restricting the development of this field [45]. Therefore, generating high-fidelity pseudo-sample data to supplement or replace real data presents a significant solution. However, the core of its feasibility lies in whether the generated pseudo-samples can enable the model to acquire generalization capabilities for real-world scenarios.

The feasibility of the method proposed in this paper is not based on theoretical assumptions but is grounded on empirical evidence from a series of experiments. Specifically, models trained solely on our synthetic data still exhibit excellent and stable detection performance on real-world datasets that were not involved in training, significantly outperforming various baseline simulated data. This demonstrates that the data generated by our method can effectively support the model in learning feature representations that are transferable to the real world. Furthermore, systematic comparisons with existing methods indicate that the physical degradation model and high-order constraints can continuously reduce the distributional discrepancy between simulated and real data. Consequently, through physically-guided generation design and empirical performance validation, this work collectively substantiates the feasibility and reliability of the proposed technical pathway in addressing the challenge of real data scarcity.

## 6. Conclusions

This paper proposes a novel pseudo-sample generation method based on physical information-driven and high-order constraints, overcoming the limitations of existing fully supervised learning approaches that heavily rely on difficult-to-annotate real infrared datasets for training. First, we developed the TOA image degradation model, which preserves reasonable information entropy distribution to create a degraded image database for pseudo-sample generation. Second, we introduced an online grid-based high-order constrained pseudo-sample generation method that integrates global target constraints, local target constraints, and local grayscale-level constraints. Through feature distribution consistency optimization, this approach ensures more reasonable target positioning, layout, and realism in generated images. Additionally, we created target position-confidence labels for network training, guaranteeing information integrity in feature representation. Finally, we established a complete network training framework that achieves successful convergence. Experimental results demonstrate that our self-supervised learning framework not only effectively suppresses complex degradation effects in real-world imaging and improves detection efficiency, but also addresses numerous challenges arising from data scarcity in deep learning applications for infrared dim and small target detection.

## Figures and Tables

**Table 1:** Quantitative comparison results of five networks trained on six simulated datasets and evaluated on the real dataset NUAA-SIRST. The gray-shaded row highlights the results of our proposed method. The best and second-best results are highlighted in red and green, respectively. Symbols ↑ and ↓ indicate that higher and lower values are better, correspondingly.

Network	Train Datasets	Pod(Pixel) ↓	F1(%) ↑	Prec(%) ↑	$F_{a}\;\left(10^{-6}\right)$ ↓	IoU(%) ↑
MDvsFA	NUDT-SIRST	0.552	80.741	94.328	6.215	81.624
	NUST-SIRST	0.405	94.215	95.743	5.322	91.842
	IRDST-simulation	9.745	67.217	74.324	20.211	61.835
	IRSTD-1K	0.468	86.844	96.745	7.417	85.328
	SIRST-5K	0.583	73.328	92.212	6.746	73.467
	ours dataset	0.342	91.745	95.328	6.219	93.433
ISNet	NUDT-SIRST	0.387	81.743	93.328	6.215	79.338
	NUST-SIRST	0.283	92.328	96.467	4.328	93.229
	IRDST-simulation	8.294	69.845	77.328	18.743	62.327
	IRSTD-1K	0.375	87.587	97.841	4.216	87.648
	SIRST-5K	0.548	74.828	91.215	8.745	74.437
	ours dataset	0.197	95.215	97.236	4.322	94.339
AFFNet	NUDT-SIRST	0.573	79.416	91.649	9.328	78.564
	NUST-SIRST	0.472	94.745	92.734	5.845	94.393
	IRDST-simulation	9.884	67.324	72.845	24.417	60.845
	IRSTD-1K	0.552	85.328	98.367	9.743	85.448
	SIRST-5K	0.615	72.417	89.328	9.216	71.206
	ours dataset	0.327	94.323	93.745	5.639	91.245
DNA-Net	NUDT-SIRST	0.372	82.323	94.744	5.843	80.215
	NUST-SIRST	0.198	95.849	97.215	3.747	92.328
	IRDST-simulation	7.845	69.747	76.213	16.828	62.434
	IRSTD-1K	0.387	88.745	97.328	3.842	87.406
	SIRST-5K	0.465	75.116	93.324	6.219	74.745
	ours dataset	0.043	92.321	98.745	2.844	95.215
RDIAN	NUDT-SIRST	0.371	83.007	96.975	4.542	82.539
	NUST-SIRST	0.112	96.884	98.735	2.755	91.447
	IRDST-simulation	8.275	69.862	77.527	16.623	64.325
	IRSTD-1K	0.335	87.613	97.304	3.492	89.106
	SIRST-5K	0.446	75.243	95.363	4.804	76.009
	ours dataset	0.036	94.305	98.841	2.614	95.386

**Table 2:** Quantitative comparison results of five networks trained on six simulated datasets and evaluated on the real dataset IRDST-real. The gray-shaded row highlights the results of our proposed method. The best and second-best results are highlighted in red and green, respectively. Symbols ↑ and ↓ indicate that higher and lower values are better, correspondingly.

Network	Train Datasets	Pod(Pixel) ↓	F1(%) ↑	Prec(%) ↑	$F_{a}\;\left(10^{-6}\right)$ ↓	IoU(%) ↑
MDvsFA	NUDT-SIRST	0.487	81.328	92.407	8.745	80.215
	NUST-SIRST	17.215	70.745	85.215	15.322	67.884
	IRDST-simulation	0.233	92.367	94.835	5.713	91.215
	IRSTD-1K	0.572	79.435	90.311	9.747	75.835
	SIRST-5K	0.387	85.745	95.205	5.479	85.545
	ours dataset	0.058	91.219	94.845	5.329	91.735
ISNet	NUDT-SIRST	0.573	80.313	90.213	10.634	82.862
	NUST-SIRST	15.416	68.437	83.205	17.145	69.954
	IRDST-simulation	0.128	94.235	96.745	3.528	92.135
	IRSTD-1K	0.673	77.417	92.339	8.745	76.267
	SIRST-5K	0.548	86.348	96.644	4.234	86.382
	ours dataset	0.157	92.864	95.706	3.228	91.015
AFFNet	NUDT-SIRST	0.412	79.215	89.348	19.449	79.463
	NUST-SIRST	18.745	67.431	81.397	18.338	65.675
	IRDST-simulation	0.022	91.369	91.615	4.739	90.396
	IRSTD-1K	0.739	76.328	89.467	9.326	75.254
	SIRST-5K	0.318	86.845	91.228	5.744	84.379
	ours dataset	0.637	90.375	92.547	7.764	89.328
DNA-Net	NUDT-SIRST	0.512	81.841	93.328	5.413	83.841
	NUST-SIRST	14.326	69.463	83.745	12.218	69.486
	IRDST-simulation	0.053	94.719	97.328	3.845	94.728
	IRSTD-1K	0.515	78.337	92.746	6.309	74.229
	SIRST-5K	0.472	87.845	97.215	3.795	87.336
	ours dataset	0.089	93.703	96.328	3.467	92.845
RDIAN	NUDT-SIRST	0.548	86.243	95.383	3.741	84.331
	NUST-SIRST	11.563	73.318	87.534	**13.365**	70.198
	IRDST-simulation	0.046	94.642	98.046	2.881	93.142
	IRSTD-1K	0.442	81.227	93.224	6.804	78.247
	SIRST-5K	0.363	89.145	97.512	3.416	87.512
	ours dataset	0.051	94.817	97.663	2.552	92.856

**Table 3:** Statistics of position pixel deviation (unit: pixel) for various models on the real dataset IRDST-real.

Model/Train Datasets	NUST-SIRST	NUDT-SIRST	IRSTD-1K	SIRST-5K	IRDST-Simulation	Ours Dataset
MDvsFA	45	15	20	20	20	12
ISNet	121	34	65	8	18	7
AFFNet	123	34	58	19	5	8
DNA-Net	456	21	33	6	5	12
RDAN	99	49	40	23	8	19
Total	844	153	216	76	56	58

**Table 4:** Qualitative comparison of five schemes on three evaluation metrics for the real datasets NUAA-SIRST and IRDST-real. The best and second-best results are highlighted in red and green, respectively. Symbols ↑ and ↓ indicate that higher and lower values are better, correspondingly.

**Scheme**	NUAA-SIRST					IRDST-Real				
	**Pod(Pixel) ↓**	**F1(%) ↑**	**Prec(%) ↑**	**$F_{a}\;\left(10^{-6}\right)$ ↓**	**IoU(%) ↑**	**Pod(Pixel) ↓**	**F1(%) ↑**	**Prec(%) ↑**	**$F_{a}\;\left(10^{-6}\right)$ ↓**	**IoU(%) ↑**
A	8.935	75.601	79.549	11.842	77.163	13.107	71.479	73.004	16.352	75.060
B	4.337	90.734	94.046	7.485	89.549	8.104	88.592	85.116	10.440	82.339
C	1.205	88.249	97.118	3.117	96.330	6.996	85.380	93.810	7.637	87.548
D	0.036	94.305	98.841	2.614	95.386	0.051	92.817	97.663	2.552	92.856

**Table 5:** Qualitative comparison of four blur degradation schemes on two real datasets and three evaluation metrics. The best and second-best results are highlighted in red. Symbols ↑ and ↓ indicate that higher and lower values are better, correspondingly.

**Scheme**	NUAA-SIRST			IRDST-Real		
	**Pod(Pixel) ↓**	**Prec(%) ↑**	**$F_{a}\;\left(10^{-6}\right)$ ↓**	**Pod(Pixel) ↓**	**Prec(%) ↑**	**$F_{a}\;\left(10^{-6}\right)$ ↓**
A	0.697	98.013	2.994	2.510	95.421	4.232
B	0.036	98.841	2.614	0.051	97.663	2.552

**Table 6:** Qualitative comparison of three Radiation Intensity Degradation schemes on two real datasets and three evaluation metrics. The best and second-best results are highlighted in red and green, respectively. Symbols ↑ and ↓ indicate that higher and lower values are better, correspondingly.

**Scheme**	NUAA-SIRST			IRDST-Real		
	**Pod(Pixel) ↓**	**Prec(%) ↑**	**$F_{a}\;\left(10^{-6}\right)$ ↓**	**Pod(Pixel) ↓**	**Prec(%) ↑**	**$F_{a}\;\left(10^{-6}\right)$ ↓**
A	2.196	96.164	3.087	1.195	96.360	3.260
B	0.171	97.913	2.726	3.997	95.191	3.793
C	0.036	98.841	2.614	0.051	97.663	2.552

**Table 7:** Qualitative comparison of three scale degradation schemes on two real datasets and three evaluation metrics. The best and second-best results are highlighted in red and green, respectively. Symbols ↑ and ↓ indicate that higher and lower values are better, correspondingly.

**Scheme**	NUAA-SIRST			IRDST-Real		
	**Pod(Pixel) ↓**	**Prec(%) ↑**	**$F_{a}\;\left(10^{-6}\right)$ ↓**	**Pod(Pixel) ↓**	**Prec(%) ↑**	**$F_{a}\;\left(10^{-6}\right)$ ↓**
A	3.650	96.684	3.441	1.237	97.586	2.603
B	2.227	97.169	2.691	1.572	96.597	5.961
C	0.036	98.841	2.614	0.051	97.663	2.552

**Table 8:** Qualitative comparison of three constraint schemes on five evaluathion metrics for the real datasets NUAA-SIRST and IRDST-real. The best and second-best results are highlighted in red and green, respectively. Symbols ↑ and ↓ indicate that higher and lower values are better, correspondingly.

Scheme			NUAA-SIRST					IRDST-Real				
$C_{g}$	$C_{l}$	$C_{\mathrm{gl}}$	**Pod(Pixel) ↓**	**F1(%) ↑**	**Prec(%) ↑**	**$F_{a}\;\left(10^{-6}\right)$ ↓**	**IoU(%) ↑**	**Pod(Pixel) ↓**	**F1(%) ↑**	**Prec(%) ↑**	**$F_{a}\;\left(10^{-6}\right)$ ↓**	**IoU(%) ↑**
✓			1.639	91.064	98.967	3.916	94.225	1.938	85.394	91.031	5.190	87.438
✓	✓		0.227	93.228	96.371	2.837	93.187	4.204	88.712	95.194	3.776	90.550
✓	✓	✓	0.036	94.305	98.841	2.614	95.386	0.051	92.817	97.663	2.552	92.856

**Table 9:** Impact of different loss balancing factor combinations $\lambda_{1}$ and $\lambda_{2}$ on self-supervised performance. “Epoch” indicates the iteration at which the model begins to converge. The best and second-best results are highlighted in red and green, respectively. Symbols ↑ and ↓ indicate that higher and lower values are better, correspondingly.

Scheme ($\lambda_{1}$, $\lambda_{2}$)	Pod(Pixel) ↓	F1(%) ↑	Prec(%) ↑	$F_{a}\;\left(10^{-6}\right)$ ↓	Epoch
(0.1, 0.9)	0.042	92.85	96.31	3.87	36
(0.2, 0.8)	0.036	94.30	98.84	2.61	23
(0.3, 0.7)	0.039	94.67	97.92	3.04	28
(0.4, 0.6)	0.045	93.95	95.76	3.78	35
(0.5, 0.5)	0.057	91.20	96.80	4.33	67
(0.6, 0.4)	0.051	88.37	94.20	5.12	65
(0.7, 0.3)	0.063	89.50	92.45	7.81	86
(0.8, 0.2)	0.075	87.40	90.10	10.25	90
(0.9, 0.1)	0.088	83.92	88.20	12.64	112

## Data Availability

The data that support the findings of this study are available from the corresponding author upon reasonable request.

## Supplementary Materials

The following supporting information can be downloaded at: https://www.mdpi.com/article/10.3390/e27121212/s1.
